# Genome-wide characterisation of HD-Zip transcription factors and functional analysis of PbHB24 during stone cell formation in Chinese white pear (*Pyrus bretschneideri*)

**DOI:** 10.1186/s12870-024-05138-w

**Published:** 2024-05-23

**Authors:** Qi Wang, Yueyang Wang, Fanhang Zhang, Chengyang Han, Yanling Wang, Mei Ren, Kaijie Qi, Zhihua Xie, Shaoling Zhang, Shutian Tao, Katsuhiro Shiratake

**Affiliations:** 1 Sanya Institute, College of Horticulture, State Key Laboratory of Crop Genetics and Germplasm Enhancement, Nanjing Agricultural University, Nanjing, 210095 China; 2 Laboratory of Horticultural Science, Graduate School of Bioagricultural Sciences, Nagoya University, Chikusa, Nagoya, 464-8601 Japan

**Keywords:** Genome-wide analysis, HD-Zip, Pear, Lignin, Stone cell, Transcription factor

## Abstract

**Background:**

The homodomain-leucine zipper (HD-Zip) is a conserved transcription factor family unique to plants that regulate multiple developmental processes including lignificaion. Stone cell content is a key determinant negatively affecting pear fruit quality, which causes a grainy texture of fruit flesh, because of the lignified cell walls.

**Results:**

In this study, a comprehensive bioinformatics analysis of HD-Zip genes in Chinese white pear (Pyrus bretschneideri) (PbHBs) was performed. Genome-wide identification of the PbHB gene family revealed 67 genes encoding PbHB proteins, which could be divided into four subgroups (I, II, III, and IV). For some members, similar intron/exon structural patterns support close evolutionary relationships within the same subgroup. The functions of each subgroup of the PbHB family were predicted through comparative analysis with the HB genes in Arabidopsis and other plants. Cis-element analysis indicated that PbHB genes might be involved in plant hormone signalling and external environmental responses, such as light, stress, and temperature. Furthermore, RNA-sequencing data and quantitative real-time PCR (RT-qPCR) verification revealed the regulatory roles of PbHB genes in pear stone cell formation. Further, co-expression network analysis revealed that the eight PbHB genes could be classified into different clusters of co-expression with lignin-related genes. Besides, the biological function of PbHB24 in promoting stone cell formation has been demonstrated by overexpression in fruitlets.

**Conclusions:**

This study provided the comprehensive analysis of PbHBs and highlighted the importance of PbHB24 during stone cell development in pear fruits.

**Supplementary Information:**

The online version contains supplementary material available at 10.1186/s12870-024-05138-w.

## Background

Transcription factors (TFs) are important regulators of gene transcription that bind to a specific nucleotide sequence upstream, which usually contains a DNA-binding domain, transactivation domain, oligomerization site, and nuclear localisation signal [1]. Genes encoding TFs are generally part of large multigene families, play an important role in controlling biological processes [2, 3]. Homeobox genes encode a homeodomain (HD) protein domain, which participates in a wide variety of developmental processes, and were first identified in Drosophila [4]. The first plant homeobox-containing gene *KNOTTED1* was identified in maize (*Zea mays*) by transposon tagging [5]. Henceforth, several Homeobox-containing genes have been extensively investigated in a wide variety of plants.

In plants, homeobox family genes are classified into 14 subclasses based on the HD sequences and the characteristic codomains: homeodomain associated to a leucine zipper (HD-Zip) I to IV, BEL, Knotted related homeobox (KNOX), zinc finger associated to a homeodomain (ZF-HD), Wuschel related homeobox (WOX), plant homeodomain associated to a finger domain (PHD finger), DDT, NDX (Nodulin homeobox genes), Luminidependens (LD), AWADEE and PINTOX [6]. Members of the HD-Zip family have a leucine zipper motif (LZ) immediately following the HD protein domain [7]. The HD and LZ motifs are present in the TFs of species in other eukaryotic kingdoms; however, their combination in a single protein is unique to plants [8]. HD-Zips have been extensively investigated and functionally characterised in many species. In *Arabidopsis thaliana*, HD-Zip is involved in several biological processes such as organ and vascular development [9], secondary cell wall (SCW) development [10], hormone action mediation [11], meristem regulation [12], and responses to environmental conditions [13].

Forty-seven HD-Zips in *Arabidopsis thaliana* [7], 63 in *Populus trichocarpa* [14], 33 in grapes (*Vitis vinifera*) [15], 43 in potato [16] have been identified. Recently, HD-Zips in Rosaceae, including apple (*Malus domestica*) [17], peach (*Prunus persica*) [18], strawberry (*Fragaria vesca*) [19], and *Prunus mume* [20] have been identified and functionally characterised. However, the distribution of HD-Zips in Chinese white pear has not been fully identified and characterised.

Pears (*Pyrus* spp.), belonging to the rose family (Rosaceae), are one of the most important fruit trees worldwide, and are cultivated in all temperate-zone countries [21]. Pear fruit is commonly eaten fresh or canned as a nutritious food, even in dietary medicine for the prevention of PM2.5 and Covid-19 among people with multiple underlying diseases [22]. Stone cells are the key determinants reducing pear fruit quality, and have been identified as thick-walled tissue cells; their formation was attributed to the thickening of the SCWs with lignin and cellulose deposition and programmed cell death [23, 24]. HD-Zip family proteins have been reported to be involved in various growth and developmental processes, such as lignification and SCW formation [25, 26], flowering [27], fruit development [28], and reponse to phytohormone and environmental signals [29, 30]. Therefore, the well-assembled genome sequence for Chinese white pear allowed us to identify and analyse HD-Zip genes at the whole-genome level to predict their function in pear stone cell formation. In our previous study, bioinformatics was used to identify HD-Zip genes in Chinese white pear, and analyse the gene structure, chromosomal location, collinearity, conserved motifs, phylogenetic relationships, and cis-acting elements of promoters. Functional prediction through comparative analysis of the HD-Zip gene in Arabidopsis helped characterise genes from other species. This study lays a solid foundation for further investigation of the biological functions of HD-Zip TFs in pears.

## Materials and methods

### Plant materials

The pear fruit samples used in this study were harvested from an orchard in Gaoyou City, Jiangsu Province, China, at different fruit developmental stages in 2021. Pear trees under uniform cultivation without disease or insect infections were randomly selected. After harvesting, the pears were transported to the laboratory at Nanjing Agricultural University (Nanjing, China). The collected samples were cut by using a sharp blade, frozen in liquid nitrogen, and then stored at -80 °C for further analysis.

### Identification of HD-Zip family members

The HD-Zip conserved domain homeodomain (PF00046) and homeobox associated leucine zipper domain (PF02183) model files were downloaded from the PFAM website (https://www.pfam.org) to obtain the Hidden Markov Model (HMM) seed file. HMMER v.3.2 software was used to search the pear protein database (http://peargenome.njau.edu.cn/) (E-value = e^− 10^) [31]. BLAST searches was performed using Arabidopsis HD-Zip genes as queries against the Chinese white pear genome databases. The redundant sequences were removed based on their identification numbers and chromosomal locations. The InterProScan program was used to confirm the presence of conserved HD-Zip domains and analysed the completeness of the HD-Zip gene domains using Pfam (http://pfam.janelia.org/) and SMART (http://smart.embl-heidelberg.de/).

### Physical and chemical properties analysis of pear HD-Zip proteins

The physicochemical properties of pear HD-Zip proteins were predicted using the ExPASy website (https://www.expasy.org/). The transmembrane structures were predicted using the TMHMM Server software (version 2.0; http://www.cbs.dtu.dk/services/TMHMM/). The subcellular locations were predicted using WOLF PSORT (https://wolfpsort.hgc.jp/) and CELLO (http://cello.life.nctu.edu.tw/).

### Multiple sequence alignments and phylogenetic relationship analysis

The amino acid sequences of HD-Zips of Chinese white pear and Arabidopsis were extracted and aligned with MAFFT v.7.4 software using default parameters [32, 33]. The HD-Zip proteins in pears were aligned and divided into different domains using the Jalview software v.2.10 [34]. ClustalX software was used to verify these results [35, 36]. The phylogenetic trees were constructed using IQ-TREE v.1.6 using the Maximum Likelihood model with 1000 bootstrap replicates. The phylogenetic treeS were visualised using the Figtree 1.4.3 software and iTOL website (https://itol.embl.de/).

### Motif composition and gene structure analysis

The conserved motifs of the PbHB genes were analysed using Motif EM for motif elicitation (MEME) v.5.0 software based on the protein sequences [37]. The chromosome location of the PbHB genes was extracted from the gene annotation files. The coding and non-coding regions and pattern of exon-introns were characterised using the Gene Structure and Display Server (GSDS) software (version 2.0; http://gsds.cbi.pku.edu.cn/index.php). Motif composition and gene structure analyses were performed using TBtools software [38].

### Gene collinearity relationships

The MCScanX (multiple collinearity scan) toolkit was used for collinearity analysis between multiple genomes and the pear genome, and homologous regions between pears and Arabidopsis were anchored. The relationships of gene collinearity were visualised using the TBtools software and the Python package circos (https://github.com/Tanghaibao/circos) [38].

### Cis-element analysis

The 2,000 bp upstream region of each PbHB gene was extracted using Tbtools software and submitted to the PlantCARE database (http://bioinformatics.psb.ugent.be/webtools/plantcare/html/) to predict cis elements [39, 40]. Sequence features of PbHBs were analysed as discribed [41, 42]. The obtained results are visualized using TBtools software [38].

### Co-expression network analysis

The expression of genes related to lignin biosynthesis in pear fruits was used to explore the expression patterns of lignin biosynthetic genes and differentially expressed HD-Zips. The expression similarity between gene pairs was characterised using Pearson’s correlation coefficient (PCC) values. The values were then filtered using Excel software (the parameter was set to > 0.6). Visualisation of the data was performed using the Cytoscape software.

### RT-qPCR analysis

RT-qPCR was performed to determine gene expression levels. The TRIzol kit provided by Tiangen Biotech (Beijing, China) Co., LTD was used to extract total RNAs from the collected plant materials, as instructed by the manufacturer. Single-stranded cDNA was synthesised using a cDNA Reverse Transcription Kit (Takara Bio, Shiga, Japan) according to the manufacturer’s instructions. RT-qPCR was conducted with the SYBR® Green Master (Roche, Basel, Switzerland) in a sequence detection system LightCycler 480 (Roche). The 2^−ΔΔCT^ method was used to calculate the relative expression levels [43, 44].

### Gene cloning, vector construction, and transient overexpression in pear fruit

The full-length of PbHB24 was cloned with primer pairs containing Xba *I* and Bam*H I* restriction sites using high performance DNA polymerase KOD FX Neo (TOYOBO, Japan). The fusion construct 35 S:: PbHB24 was generated by inserting the gene fragment into the pCAMBIA1300 vector using the One-step Rapid Cloning Kit. Then, The plasmids of empty vector and 35S:: PbHB24 fusion constructs were transferred to GV3101 according to its illustration. Agrobacterium cells were injected into the flesh of ‘Dangshansuli’ fruits at 35 Days after full bloom (DAFB) using needleless syringes as discribed previously [45]. Twenty fruits were injected with each construct. Samples were collected 7 days after injection. Stone cell contents and acetyl bromide lignin contents of pear fruit were measured as described before [24, 45]. Stone cell and lignin measurements were replicated for at least six biological replicates.

## Results

### Identification, genome distribution, and collinearity of HD-Zip members in Chinese white pear

**Fig. 1:**
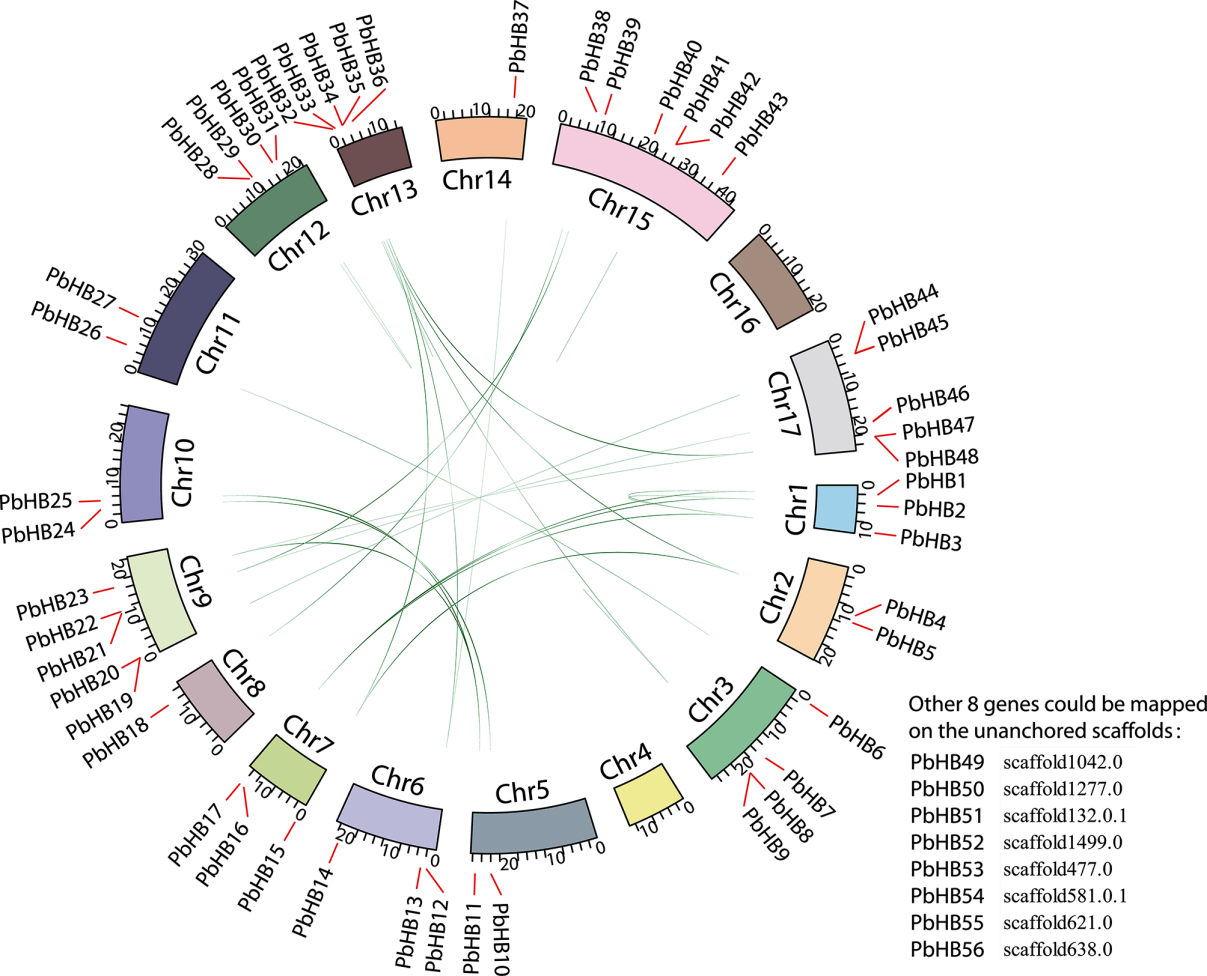
Genome distribution and collinearity of the pear HD-Zip family. HD-Zip genes are mapped to pear chromosomes by the TBtools. Pear chromosomes arranged in a circle. Red lines mark the position of genes on chromosomes. Blue lines indicate a collinear relationship among genes

We searched the entire Chinese white pear genome for genes that encode proteins with the HD-Zip conserved domains. Ultimately, 56 *PbHB* genes were identified using Pfam and interProScan, after manually checked. Among these 56 PbHB genes, 48 genes were unevenly distributed on the chromosomes, and the other eight genes could be mapped to the unanchored scaffolds (Fig. 1, Table S1). The molecular weights (MW) of these PbHB genes ranged from 19505.22 to 114045.3 Da. The predicted isoelectric points range from 4.61 to 9.46. Gene subcellular localization prediction indicated that all the PbHB proteins were possibly located in the nucleus (Table S1).

Based on their position on the chromosomes, the HD-Zip genes in pears were denominated as *PbHB1*-*PbHB56* (Table S1). As shown in Fig. 1, no PbHB gene was located on chromosome 4 or 16. Chromosomes 1 and 13 are short but contain three and five PbHB genes, respectively. A few PbHB genes were well-proportioned and mapped to one chromosome but were mostly distributed on certain fragments of chromosomes (Fig. 1). This may be attributed to uneven duplication events in pear chromosome fragments. The evolution of the PbHB family has been promoted by different gene duplication modes, including whole-genome duplication (WGD), tandem duplication (TD), transpose duplication (TRD), and dispersed duplication (DSD). All PbHB genes were assigned as WGD, TD, TRD, or DSD. Among the duplication events, DSD played a significant role in the evolution of PbHB (Table S2).

### Multiple alignment, classification, and sequence features of PbHBs

**Fig. 2:**
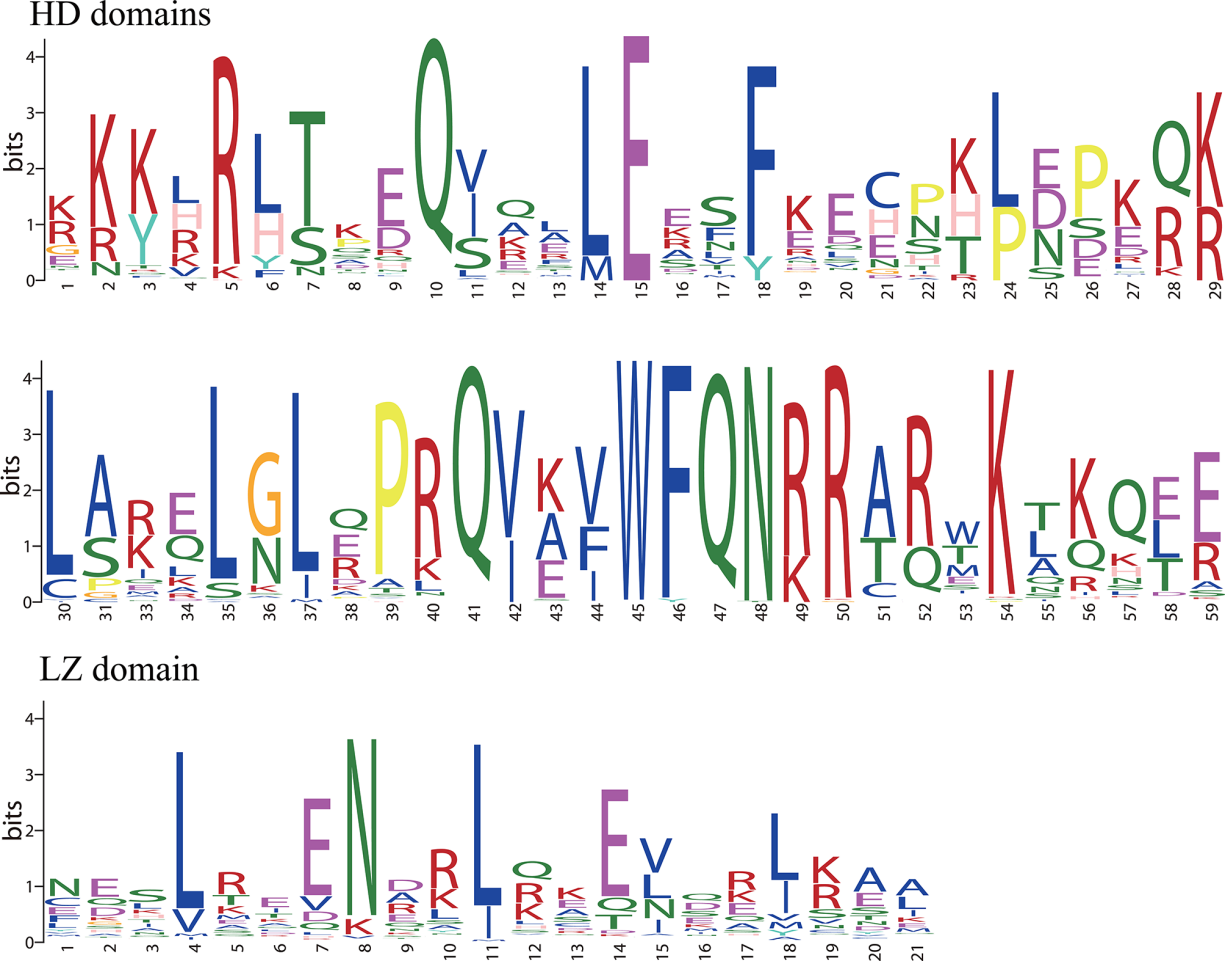
HD- and LZ domains are highly conserved across all HD-Zip proteins in the pear genome. Sequence logos of the HD- and LZ-domain are based on the sequence alignments of 56 PbHB proteins. Bit score indicates the information content for each position in the sequence. Multiple alignment analysis of 56 PbHB proteins was performed with ClustalW (see Supplementary Fig. S1)

Multiple alignment analysis was performed to investigate the homologous domain sequence features of the 56 HD-Zip proteins in pears (Fig. S1). The frequencies of the most widespread amino acids in the HD and LZ-domain were obtained (Fig. 2). The predicted HD and LZ-domain are highly conserved across plant lineages. The HD domain is responsible for DNA binding, and LZ is located immediately at the C-terminus of HD and is involved in protein-protein interactions. The results showed the basic regions of the HD and LZ domains in Chinese white pear.

**Fig. 3:**
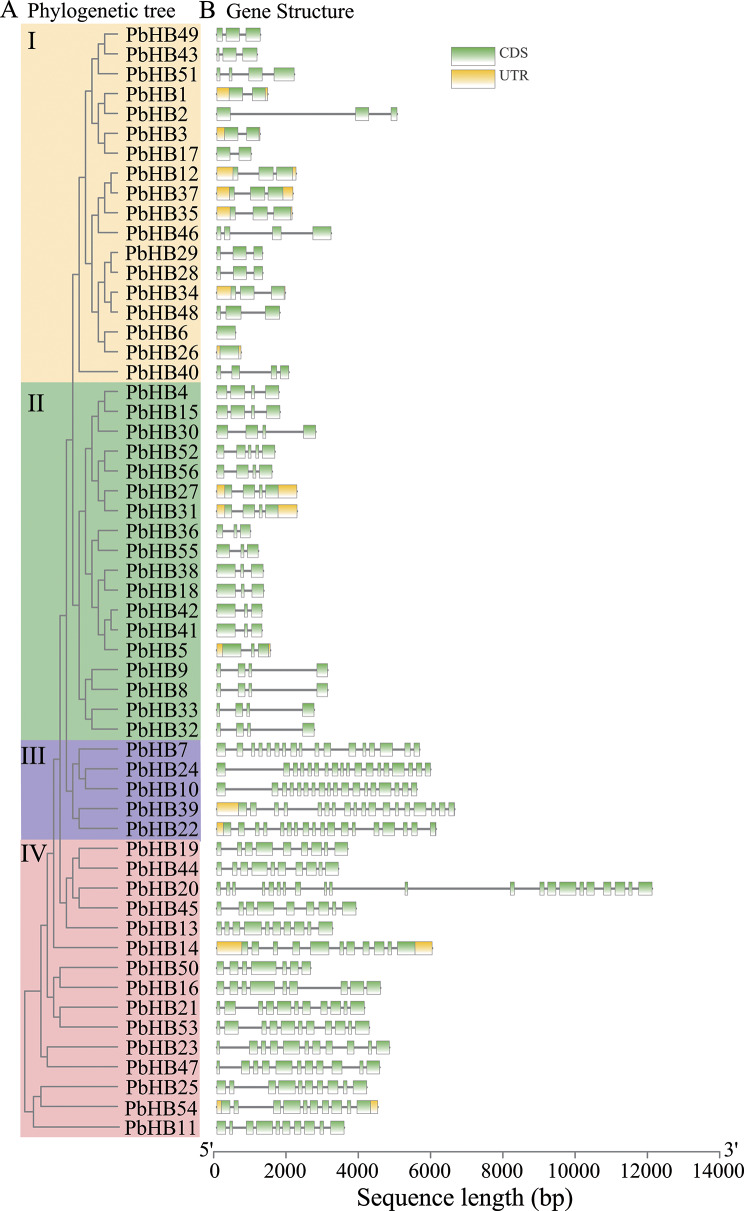
Phylogenetic relationships and gene structures of PbHBs. (**A**) Phylogenetic tree of 56 PbHB proteins. Phylogenetic tree was created in IQ-TREE v.1.6 using the Maximum Likelihood model with 1,000 bootstrap replicates. (**B**) Gene structure of PbHB family TFs in Chinese white pear. Abscissa represents the length of the nucleic acid sequence (bp)

To further investigate the phylogenetic relationships between PbHB proteins, a phylogenetic tree was constructed, which revealed that the 56 PbHB proteins of Chinese white pear could be divided into four subfamilies:18, 18, 5, and 15 members, which were identified separately in subfamilies I, II, III, and IV, respectively (Fig. 3A). Overall, 10 motifs were detected in the PbHB proteins. In the same subfamily, the number and types of conserved motifs were similar, whereas those of PbHBs were diverse in different subfamilies. Similar intron/exon structural patterns were observed in the same type of genomic structures of the 56 PbHB genes, strongly supporting their close evolutionary relationship (Fig. 3B). The exon numbers of subfamilies III and IV were much higher than those of subfamilies I and II. All the PbHBs contained at least one exon, with a maximum of 21 exons (Table S3).

### Phylogenetic relationships of HD-Zip proteins in Rosaceae and Arabidopsis

To determine the evolutionary relationships of the HD-Zip family in Rosaceae, a phylogenetic tree was constructed using the protein sequences of six Rosaceae species: 56 HD-Zip proteins from *P. bretschneideri*, 61 from *Pyrus communis*, 80 from *Malus domestica*, 33 from *Prunus persica*, 28 from *Fragaria vesca*, 42 from *Prunus mume* and 47 from Arabidopsis (Fig. S2, Table S4). They were divided into four subfamilies (I, II, III, and IV). Among the six Rosaceae species, *P. persica* and *P. mume* belong to Prunoideae, *F. vesca* belongs to Rosoideae, and *P. bretschneideri*, *P. communis*, and *M. domestica* belong to the Maloideae. A recent duplication event in Maloideae, but not in Prunoideae or Rosoideae, probably contributed to the expansion of the HD-Zip gene family in Maloideae (Fig. 3; Table 1). Notably, the HD-Zip genes of all subfamilies were duplicated in Maloideae except for the pear subfamily III. It is currently unknown whether this is the cause of stone cell occurrence in pear fruits.

**Table 1:** The number of subfamilies of HD genes in different species

Species	Total gene number	Gene number			
		I	II	III	IV
Pyrus bretschneideri	56	18	18	5	15
Pyrus communis	61	24	15	7	15
Malus domestica	80	31	21	9	19
Prunus persica	31	11	8	4	8
Fragaria vesca	28	11	6	4	7
Prunus mume	36	14	9	4	9
Arabidopsis thaliana	47	17	9	5	16

### Comparison analysis reveals the putative functions of pear HD-Zip TFs

**Fig. 4:**
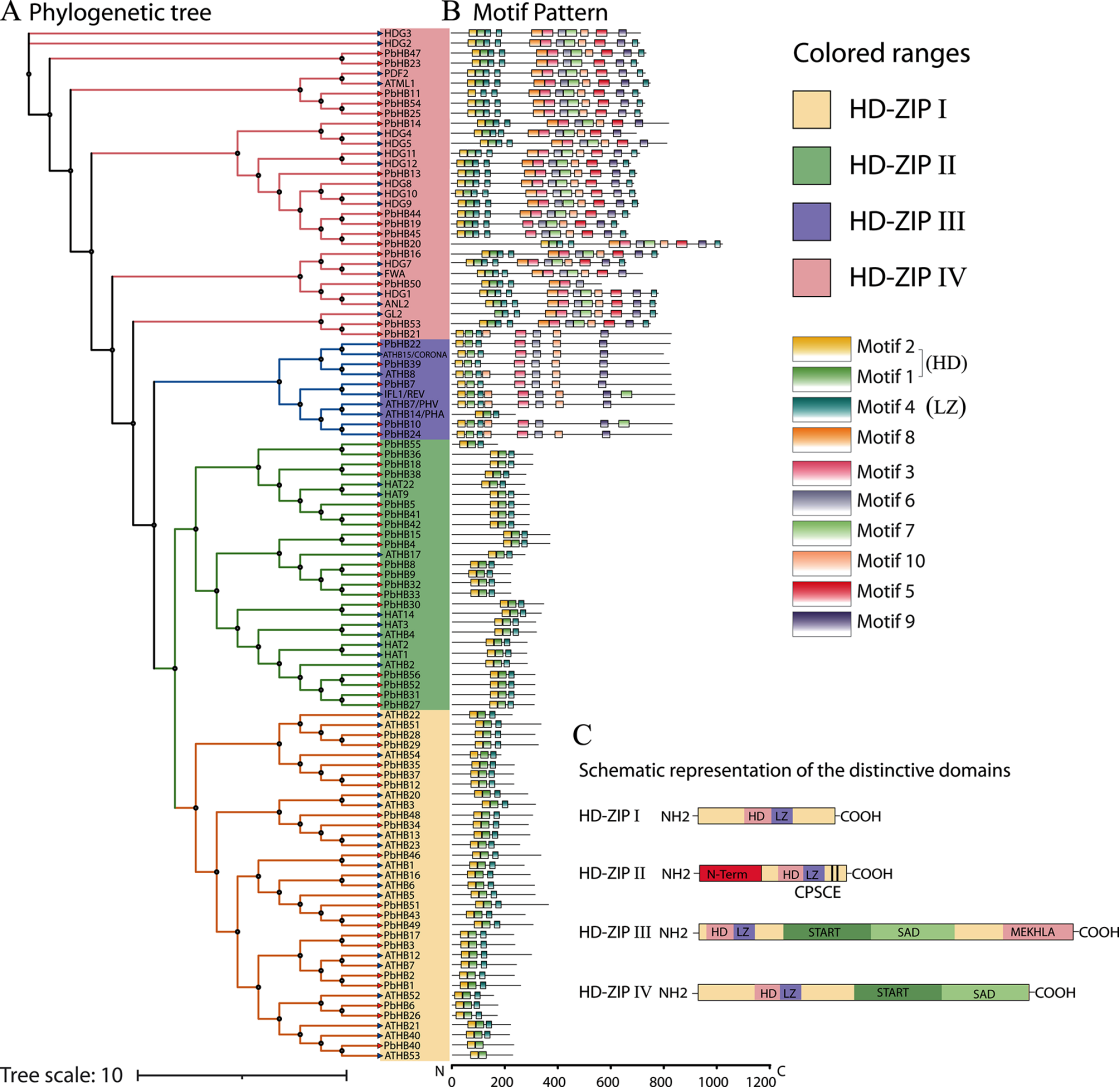
Phylogenetic analysis and motif compositions of HD-Zips from pear and Arabidopsis. (**A**) Phylogenetic tree of HD-Zip proteins from pear and Arabidopsis was constructed with IQ-TREE v.1.6 software. PbHBs were clustered into 4 distinct clades, marked by curves of different colours. (**B**) Schematic representation of the conserved motifs of the PbHBs identified by MEME. Each motif is indicated by a coloured box numbered at the bottom. Abscissa represents the amino acid length (**C**) Schematic representation of the distinctive domains exhibited by each subfamily of HD-Zip proteins. Abbreviations: MEKHLA domain, named after the highly conserved amino acids Met, Glu, Lys, His, Leu, Ala; N-term, N-terminus consensus; SAD, START adjacent domain; START, steroidogenic acute regulatory protein-related lipid transfer domain

Orthologous and paralogous analyses are indispensable in comparative genomics. Through evolutionary tree analysis, homologous genes clustered in the same branches and sub-branches often have similar functions [46]. To date, the most extensive quantitative evaluation of plant HD-Zip genes has been conducted in Arabidopsis. Therefore, a phylogenetic tree of *P. bretschneideri* and Arabidopsis was constructed to classify the functions of PbHBs based on well-characterised HD genes in Arabidopsis. The clusters of orthologous and paralogous genes was identified, which helped characterise each subfamily of the PbHB gene family (Fig. 4A). Proteins within a subfamily sharing the same motif are likely to exhibit similar biological functions. Motif composition analysis showed that HD-Zip genes were remarkably conserved between pears and Arabidopsis, indicating their similar functions (Fig. 4B). Subfamilies I and II were similar, containing only the HD and LZ-domain, whereas subgroups III and IV were significantly different, containing multiple other motifs (Fig. 4B). Subgroups with specific motifs may also have specific functions.

There are few reports on the biological functions of HD-Zip proteins in other model plants, such as poplar, rice, and tomato. In this study, the biological functions of HD-Zip genes were characterised by homology comparison (Table 2). As shown in Table 2, HD-Zip I proteins are involved ABA response, fruit ripening, lignification, thermotolerance, blue-light signalling, salinity tolerance, and source-to-sink partitioning; HD-Zip II proteins are involved in shade avoidance, auxin response, illumination, ABA signalling, and gynoecium development; HD-Zip III proteins are involved in cambium and meristem formation, vascular development, SCW development, auxin biosynthesis, signalling, and transport, abscission, JA-isoleucine biosynthesis, brassinosteroid synthesis, and lignin biosynthesis; HD-Zip IV proteins are involved in lignification, specification of the epidermis, cell elongation, gibberellic acid signalling, floral organ identity, flowering phenotype, flowering time and fertility, drought tolerance, glandular trichome initiation and cuticle development, flag leaf development, and trichome elongation. The particular orthologous and paralogous proteins in pears were identified andtheir functions were characterised (Table 2). Results revealed that PbHB17, PbHB7, PbHB22, PbHB13, PbHB39 might be involved in SCW thickening and lignin formation because their homolog genes in Arabidopsis and other plants are known to play crucial roles in the regulation of SCW thickening and lignin formation; PbHB1, PbHB2, PbHB3, PbHB15, PbHB56, PbHB52, PbHB31, PbHB27, PbHB11, PbHB54, PbHB25, and PbHB39 might be involved in plant hormone signalling; and PbHB1, PbHB2, PbHB13, PbHB56, PbHB52, PbHB31, PbHB27 might be involved in stress response (Table 2). This result enhances our understanding of the putative roles of PbHB proteins.

**Table 2:** Putative functions prediction of PbHBs

Subfamily	Gene	Best hit in *P*. bretschneideri	Functions	Species	References
HD-Zip I	ATHB6/7/12	PbHB1/2/3/15	Response to ABA	Arabidopsis	[47, 48]
	VAHOX1	PbHB37	Fruit ripening	Tomato	[28]
	EjHB1	PbHB17	Lignification	Eriobotrya japonica	[49]
	LlHB16	Loss	Thermotolerance	Lilium longiflorum	[50]
	ATHB16	PbHB51/43/49	Blue-light signaling	Arabidopsis	[51]
	MdHB7	PbHB1/2	Salinity tolerance	Malus domestica	[52]
	AtHB5	PbHB51/43/49	Source-to-sink partitioning	Arabidopsis	[53]
HD-Zip II	ATHB2	PbHB56/52/31/27	Shade avoidance	Arabidopsis	[54]
	HAT2	PbHB56/52/31/27	Auxin response; Response to illumination	Arabidopsis	[55, 56]
	CaHAT1	PbHB56/52/31/27	Abscisic acid signalling	Capsicum annuum	[57]
	HAT3/ATHB4	PbHB56/52/31/27	Gynoecium development	Arabidopsis	[58, 59]
HD-Zip III	PtrHB4	PbHB10	Cambium formation	Populus	[60]
	PtrHB7	PbHB39	Vascular cambium differentiation	Populus	[61]
	PtoHB7	PbHB39	Auxin-mediated secondary xylem formation	Populus	[62]
	PHB/REV	PbHB7	Auxin biosynthesis, signaling, and transport	Arabidopsis	[63, 64]
	SlHB15A	PbHB19	Abscission; JA-isoleucine biosynthesis;	Solanum lycopersicum	[65]
	OsTFL1L	Loss	Lignin biosynthesis; Stomatal closure	Rice	[66]
	ATHB8	PbHB39	Brassinosteroid synthesis; Vascular system development	Arabidopsis	[12, 67]
	ATHB15	PbHB22	Secondary cell wall development; Vascular system development	Arabidopsis	[26, 68]
	REV	PbHB7	Lignin biosynthesis; Meristem initiation	Arabidopsis	[69, 70]
	popREVOLUTA	PbHB7	Secondary vascular development	Populus	[71]
	REV/ PHB/ PHV	PbHB7	Embryogenesis; Secondary wall synthesis	Arabidopsis	[25, 72]
HD-Zip IV	Roc8	PbHB13	Lignification.	Rice	[73]
	ATML1/PDF2	PbHB11/54/25	Specification of the epidermis; Drive cell elongation in response to gibberellic acid signaling; Endoreduplicated giant cells; Floral organ identity	Arabidopsis	[74–77]
	FWA	PbHB16	Flowering phenotype	Arabidopsis	[78]
	HDG11/12	PbHB13	Drought tolerance; Zygote polarity	Arabidopsis	[79]
	AaHD8	PbHB50	Glandular trichome initiation and cuticle development	Artemisia annua	[80]
	Roc5	Loss	Flag Leaf Development	Rice	[81]
	SlHD8	PbHB13	Trichome elongation	Tomato	[82]
	CsGL2-LIKE	AtGL2	Flowering time and fertility	Cucumber	[27]

### Cis-regulatory element assessment analysis of PbHBs

**Fig. 5:**
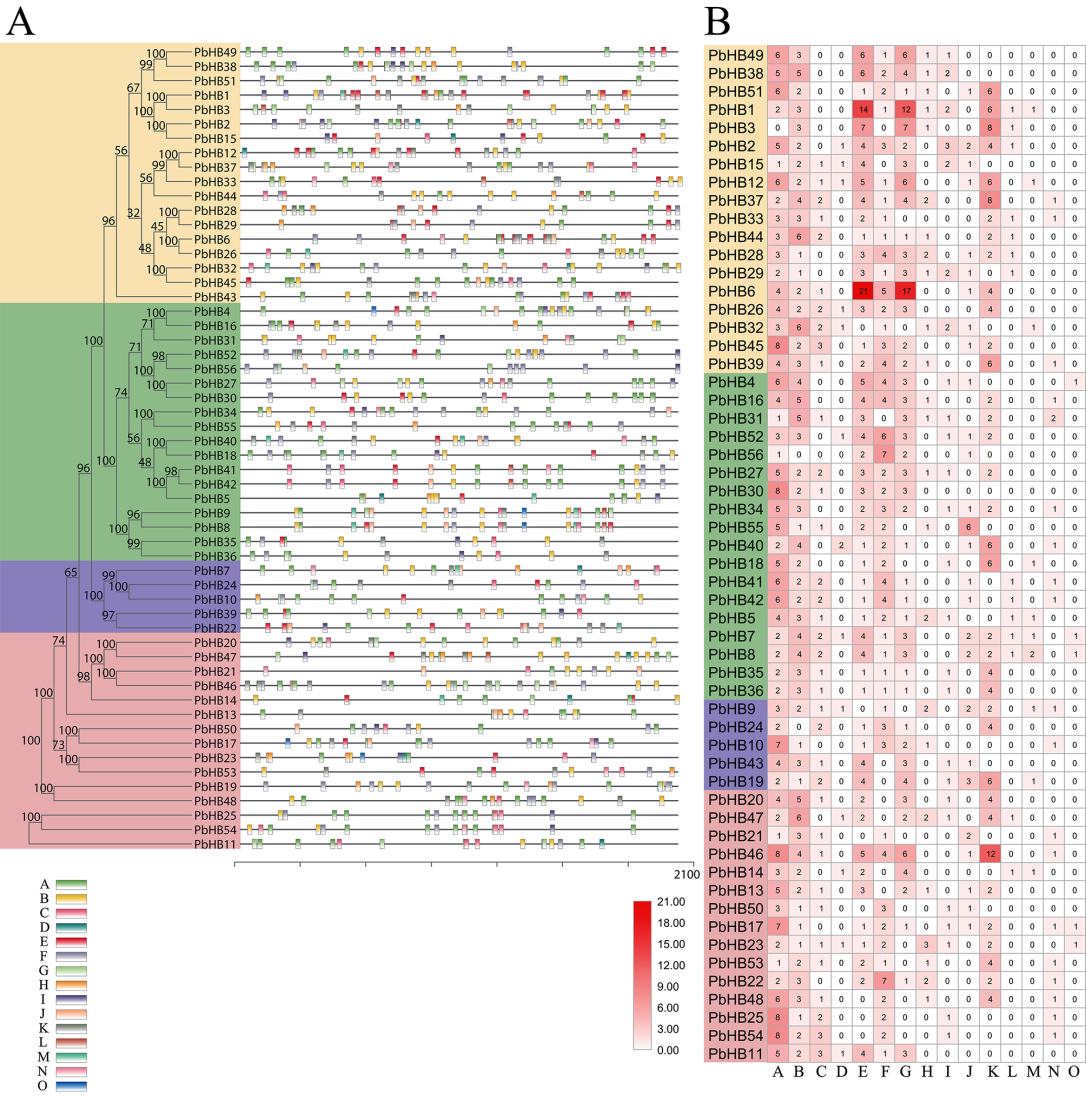
Cis-acting elements analysis of the promoter of PbHB genes. (**A**) Distribution of 15 cis-acting elements in the promoters of 56 PbHB genes. Abscissa represents the promoter sequence length. Scale bar indicates 300 bp. Different coloured boxes indicate different types of cis-acting elements. (**B**) Number of 15 cis-acting elements in promoters of 56 PbHB genes. The colour of the box indicates the number of cis-acting elements

To reveal the biological processes in which PbHB genes might be involved, the 2,000 bp upstream region of all PbHB genes was extracted to identify the cis-acting elements. After prediction using the PlantCARE website, some elements with unknown functions were eliminated, and 15 important elements were selected for further analysis. The distribution of the promoter elements in the four subfamilies is shown in Fig. 5. Overall, the number and distribution of the light-responsive components were the highest (Fig. 5A). All the PbHB genes contain light-responsive elements (Fig. 5B). Second, some hormone signals are also widely distributed in the PbHB promoters, such as MeJA responsiveness, abscisic acid responsiveness, and salicylic acid responsiveness, indicating that PbHB genes play a critical role in the response to plant hormones. Moreover, several stress-related elements were detected, including low-temperature responsiveness, defence, and stress responsiveness (Fig. 5A, B). These results indicate that PbHB genes may be involved in plant hormone signalling and external environmental responses such as light, temperature, and stress.

### Expression profiling of HD-Zips in pear fruits

**Fig. 6:**
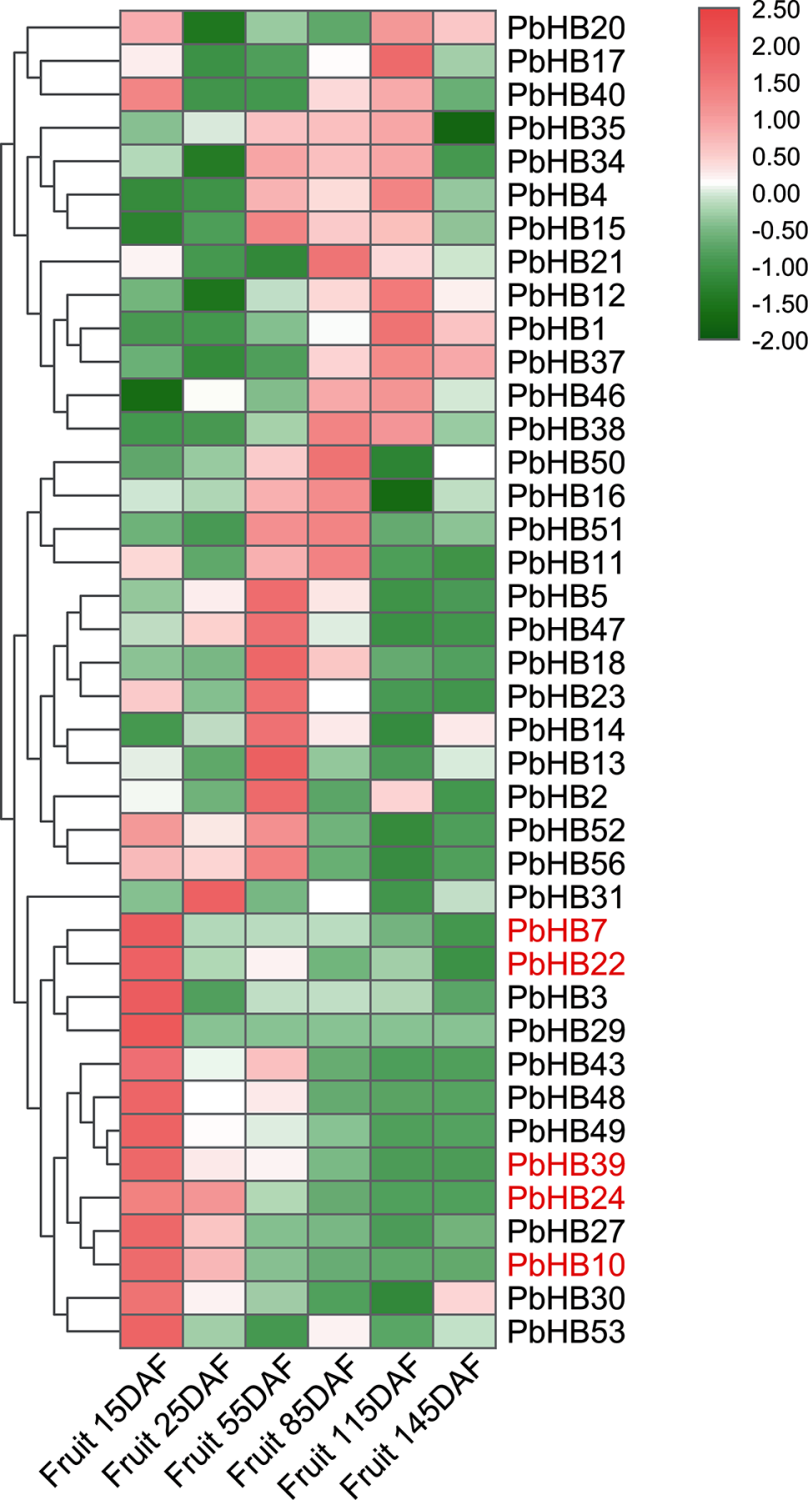
Heatmap indicates the expression pattern of HD-Zip genes in pear fruits. Gene expression of 40 PbHB genes was scaled according to the bar in colour that measures Z-scores of mean FPKM values. Five genes indicated in red means HD-Zip genes of subfamily III. Detailed information has listed in Table S5. Red represents up-regulated and green represents down-regulated. DAF, days after flowering

Stone cell content increases rapidly during the early stages of pear fruit development and is discontinued after the middle stage of development (~ 55 days after flowering) [83]. Based on RNA-sequencing data, 40 HD-Zip genes were expressed during fruit development (Fig. 6, Table S5). Fourteen of these genes were highly expressed during the early stages of fruit development, indicating their putative roles in stone cell formation. It is worth noting that all HD-Zip genes of subfamily III were specifically highly expressed in the early stages, indicating their crucial role in stone cell development.

### Co-expression network analysis of PbHB genes and lignin biosynthetic genes

**Fig. 7:**
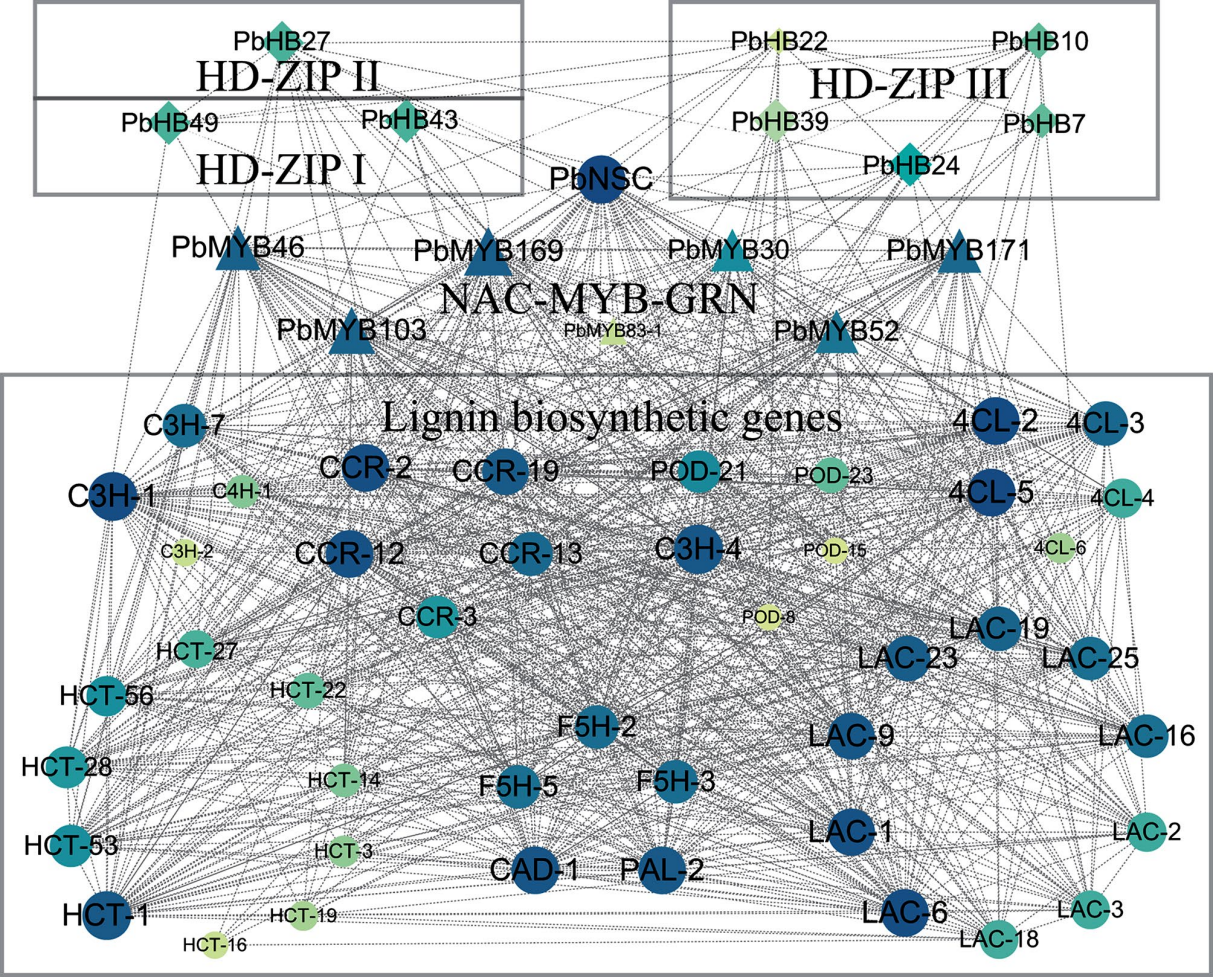
Co-expression network of *PbHBs* with lignin-related genes. RNA-seq data were used to measure the expression similarity between gene pairs as PCC values. Detailed information is listed in Table S6. Size and colour depth of each object represent degree values measured by CytoNCA [84]. The values were then filtered with Excel software (with the parameter set as > 0.6). Data were visualised using Cytoscape software

Co-expression network analysis was performed to elucidate the relationship between *PbHB* genes and lignin biosynthetic genes. Eight *PbHB* genes were classified into different co-expression clusters with lignin-related genes, including *PbHB27*, *PbHB49*, *PbHB43*, *PbHB22*, *PbHB10*, *PbHB39, PbHB7, and PbHB24* (Fig. 7; Table S6). These PbHBs were highly correlated with genes involved in lignin biosynthesis, which are highly expressed in the early stages of fruit development. Of these *PbHB* genes, *PbHB24* showed the highest correlation, indicating that it is a putative regulator of stone cell development.

### RT-qPCR analysis

**Fig. 8:**
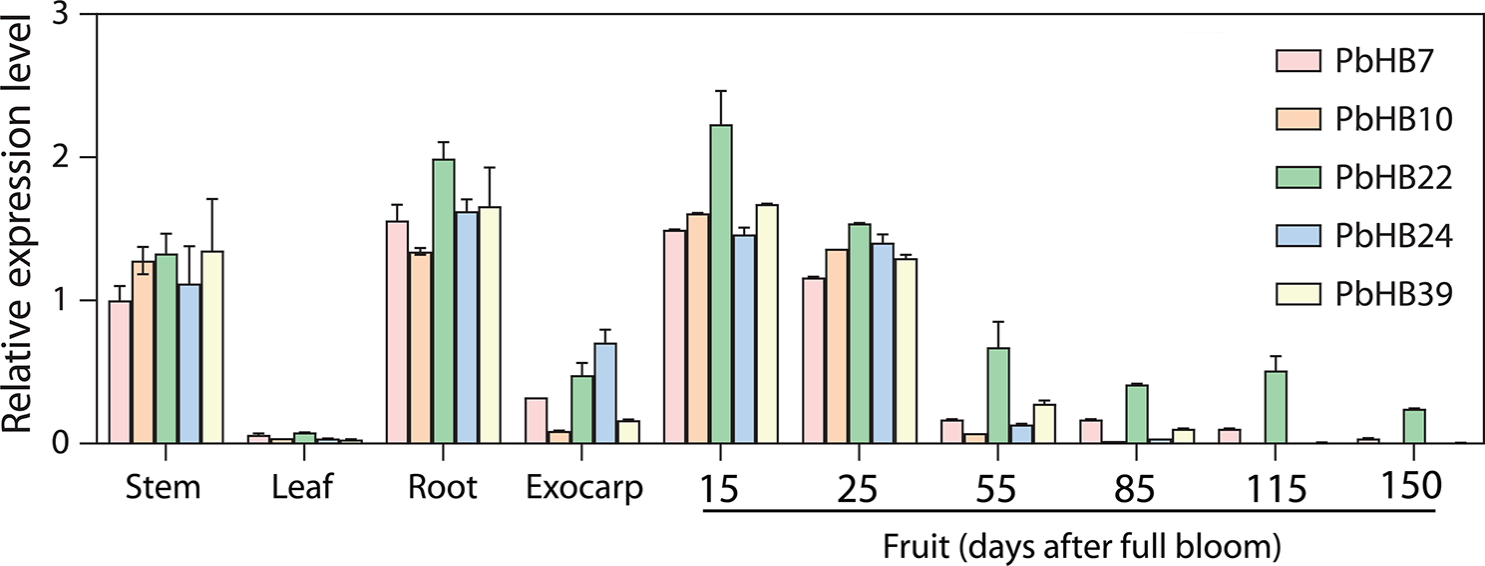
Tissue-specific expression of pear HD-Zip III genes. Primer sequences are listed in Table S7. Bar values represent the mean ± SD of three biological replicates. Different coloured bars represent different genes

The high conservation of the HD-Zip III genes throughout evolution indicates that they likely play a significant role in lignin biosynthesis and SCWs thickening. To reveal the potential role of HD-Zip III genes in pears, we monitored the expression of five HD-Zip III genes (*PbHB7*, *PbHB10*, *PbHB22*, *PbHB24*, and *PbHB39*) in different pear tissues and fruits of *D. angshansuli* at different developmental stages (Fig. 8). They were all highly expressed in the lignified tissues of pear trees, including the roots, stems, and early flesh. In the later stages of fruit development, their expression levels were extremely low, except for *PbHB22*. Stone cell formation occurs in the early stages of fruit development. A total of 188 genes involved in lignin biosynthesis were identified in the transcriptome data, which showed that they were highly expressed at two early periods of pear fruit development compared to other developmental periods (Table S6). Here, the expression trends of these HD-Zip genes in fruits correlated with stone cell development, indicating their regulatory role in stone cell formation.

### Overexpression PbHB24 promotes stone cell formation in pear fruits

**Fig. 9:**
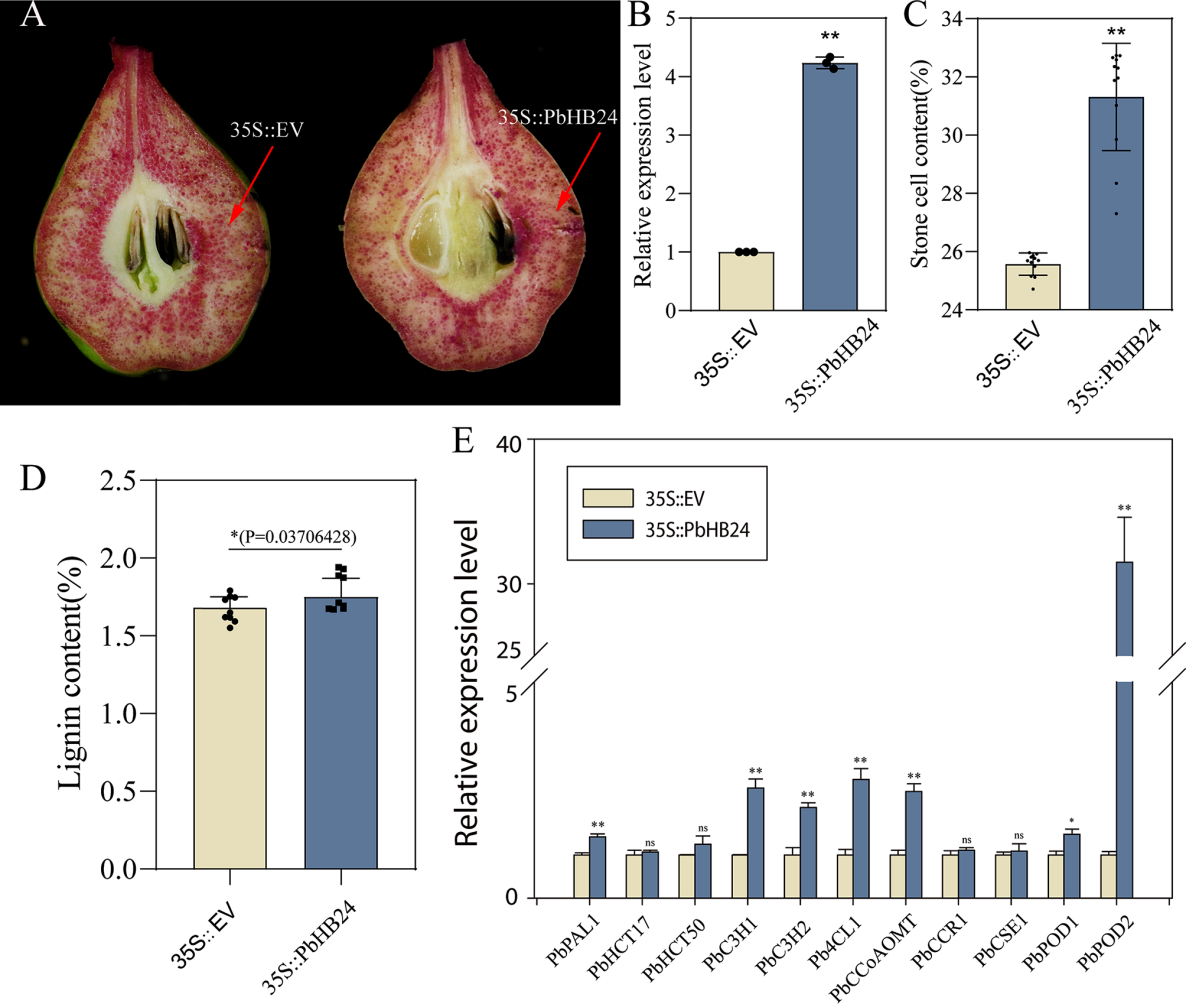
Transient overexpression of *PbHB24* in pear fruitlets. (**A**) Phloroglucinol staining of the pulp of ‘Dangshansuli’ fruitlets after *Agrobacterium*-mediated overexpression. 35 S::EV, fruitlets injected by empty vector as a control; 35 S::PbHB24, *PbHB24* overexpression vector mediated by 35 S strong promoter. (**B**) Relative expression of *PbHB24* 7 days after injection. The error bars are the means ± SD (*n* = 3). (**C**) Stone cell content of pear fruits 7 days after injection. The error bars are the means ± SD (*n* = 12). (**D**) Acetyl bromide lignin content of pear fruits 7 days after injection. The error bars are the means ± SD (*n* = 6). The vertical bars are the means ± SD of biological replicates and asterisks indicate significant differences by two-tailed Student’s *t*-test (*P < 0.05, **P < 0.01)

To verify the function of *PbHB24* in stone cell formation, the transient overexpression of *PbHB24* was conducted in ‘Dangshansuli’ at 35DAFB. Increases in lignin staining were observed at the injection sites under the overexpression of *PbHB24* 7 days after injection (Fig. 9A). RT-qPCR showed that *PbHB24* was successfully overexpressed at the 35 S::PbHB24 injection sites (Fig. 9B). The stone cell and lignin contents of overexpression sites were also increased compared with fruits injected with the control vector (Fig. 9C, D). Furthermore, the expression of multiple lignin biosynthetic genes were upregulated after *PbHB24* overexpression (Fig. 9E). These data indicate the positive regulatory role of *PbHB24* in stone cell formation in pear fruits.

## Discussion

Plants produce lignin and thickened SCWs to equip plants with mechanical strength for erect growth and the capacity for long-distance conduction. The stone cells of pear fruits negatively affect fruit quality because of their lignified cell walls [24, 85]. Thus far, knowledge of the molecular mechanisms involved in stone cell formation is limited. The development of stone cells begins with differentiation of the lateral meristem and vascular cambium into secondary xylem mother cells, followed by cell expansion, secondary wall deposition, programmed cell death, and stone cell formation. Emerging evidence suggests that HD-Zip genes are involved in the vascular cambium, secondary wall deposition, and lignin biosynthesis (Table 2). This study was conducted through the identification, gene structure, and expression analysis of the HD-Zip family members to reveal their potential role in stone cell formation.

HD-Zip protein plays a wide range of roles in plant development, including vascular development, organ formation, meristem maintenance, stress response, and environmental and hormone signalling responses. Analysis of the promoter elements of the 56 PbHB genes revealed that environmental and plant hormone-responsive elements were most widely distributed in the promoters of pear HD-Zip genes. HD-Zip II TF *HAHB10* participates in the induction of flowering and control of phytohormone-mediated responses to biotic stress in sunflowers [86]. The genes in HD-Zip II are mostly known for their functions in shade avoidance and light environment responses [87, 88]. PIF4 and the miR166-HB15 module modulate vascular development, SCW thickening, and consequently, stem-lodging susceptibility at elevated temperatures [26]. In loquat fruit, the EjbHLH14-EjHB1-EjPRX12 module is involved in the methyl jasmonate alleviation of chilling-induced lignin deposition [49]. These studies confirmed that HD-Zip genes might be crucial molecular hubs for connecting plant environmental/plant hormone signals with plant developmental signals.

In many plants, gene co-expression networks are increasingly being used to predict gene function and search for speculative targets of TFs [89]. Candidate genes involved in the regulation of anthocyanin and organic biosynthesis were prioritised based on co-expression analysis [90]. In grapes, the gene co-expression database VTCdb (http://vtcdb.adelaide.edu.au/Homethroat) offers an online platform for transcriptional regulatory inference [91]. Here, we predicted that HD-Zip III genes are critical regulators of lignification in stone cell formation, based on co-expression analysis. A previous study in Arabidopsis and the high conservation of HD-Zip III genes indicated that it likely plays a significant role in lignin biosynthesis and SCWs thickening. In Arabidopsis, *miR165/166* and its downstream targets, five HD-Zip III genes, play conserved roles in vascular development and SCW formation in vascular plants [92]. HD-Zip III genes are characterised by an HD-Zip domain for DNA binding and protein dimerisation, and a highly conserved lipid or steroid-binding steroidogenic acute regulatory protein-related lipid transfer (START) domain. The two NAC master switches, *SND1* and *NST2*, are upregulated in *athb15* mutants, confirming that *AtHB15* functions as a negative regulator of secondary wall-related regulatory pathways [10]. REV promotes lignin biosynthesis by binding to the promoter of the lignin biosynthesis gene *PHENYLALANINE AMMONIA LYASE4* (*PAL4*) [69]. These studies demonstrate that HD-Zip III genes play a crucial role in lignification; however, knowledge of their specific molecular network is still limited.

During secondary growth, xylem and phloem are further expanded via the differentiation of cells derived from division in the cambium. Almost all developmental fate decisions, including vascular specification, patterning, and differentiation, are regulated by TFs belonging to the HD-Zip III family. TFs are important regulators of gene transcription that bind to specific upstream DNA sequences [1]. HD-Zip I genes are generally encoded by 35 kDa proteins and exhibit a highly conserved HD domain, a less conserved LZ domain, and no other similarities. PCR-assisted binding-site selection and footprinting assays revealed the ability of HD-Zip I proteins to recognise and bind to the pseudopalindromic sequence [CAAT(A/T)ATTG] [93]. The HD-Zip II and III binding sites share the same core sequence [AAT(G/C)ATT] [88, 94]. HD-Zip class IV TFs bind to the L1BX-like sequence, [TAAATG(C/T)A] [95]. These reports establish a solid foundation for studying HD gene regulation during lignification and SCWs thickening. Future studies should focus on identifying the molecular pathways through which the PbHB genes regulate lignin biosynthesis in stone cells.

### Electronic supplementary material

Below is the link to the electronic supplementary material.

Supplementary Material 1

Supplementary Material 2

Supplementary Material 3

Supplementary Material 4

Supplementary Material 5

Supplementary Material 6

Supplementary Material 7

Supplementary Material 8

Supplementary Material 9

## Data Availability

The datasets analyzed during the current study are included within the article and its supplementary information fIles. The RNA sequencing data is available in the NCBI BioProject database (https://www.ncbi.nlm.nih.gov/bioproject.) under the accession numbers PRJNA825067 and The Pear Expression Database (http://www.peardb.org.cn/).

## Abbreviations

HD-Zip: Homodomain-leucine zipper
RT-qPCR: Quantitative real-time PCR
TFs: Transcription factors
KNOX: Knotted related homeobox
ZF-HD: Zinc finger associated to a homeodomain
PHD: Plant homeodomain associated to a finger domain
LD: Luminidependens
LZ: Leucine zipper motif
SCW: secondary cell wall
MEME: Motif EM for motif elicitation
WGD: Whole-genome duplication
TD: Tandem duplication
TRD: Transpose duplication
DSD: Dispersed duplication
