# Supplementary figures and images for: Genome-wide characterisation of HD-Zip transcription factors and functional analysis of PbHB24 during stone cell formation in Chinese white pear (Pyrus bretschneideri)
Source: BMC Plant Biol. 2024 May 23;24:444. doi: 10.1186/s12870-024-05138-w (PMC11112822; doi:10.1186/s12870-024-05138-w)

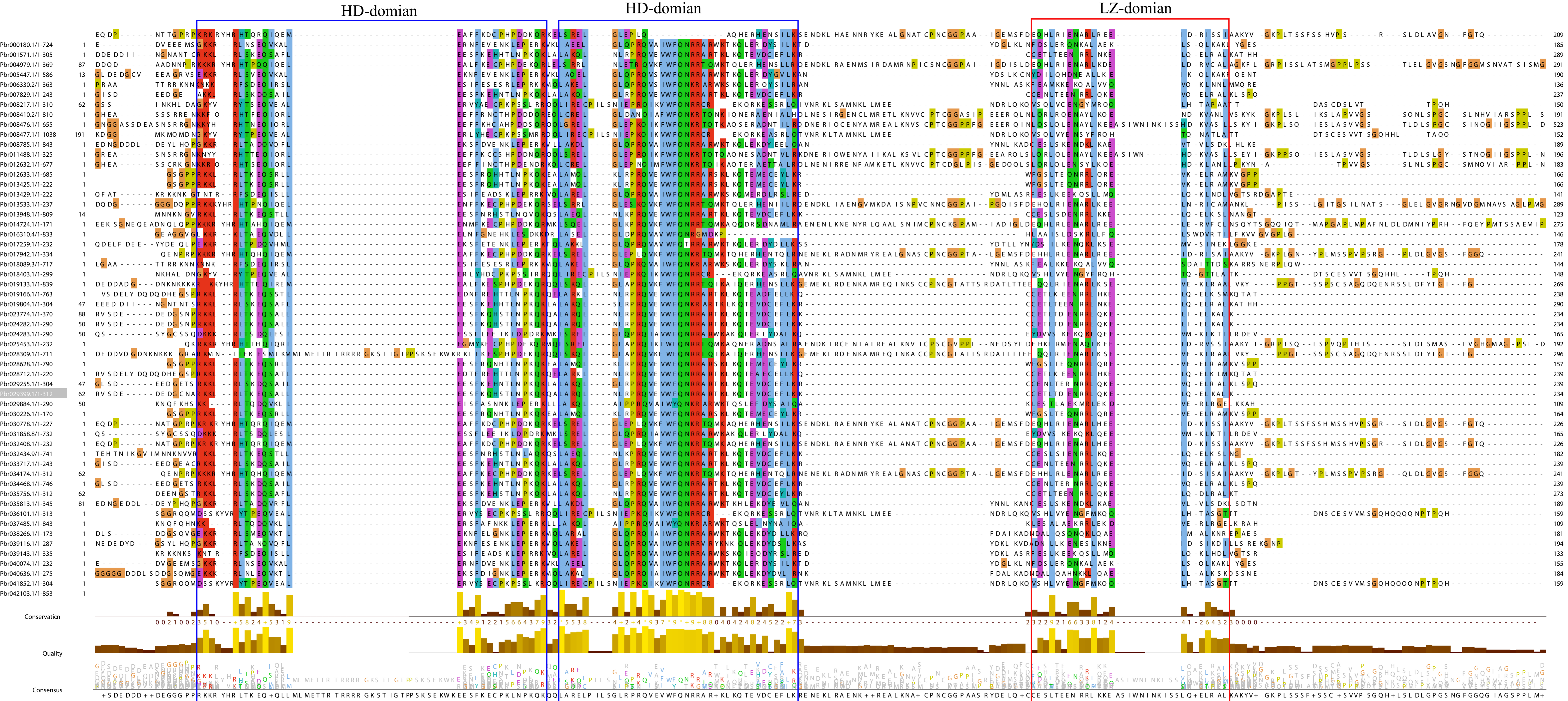

Supplement: Supplementary file 8 — Supplementary Material 8 [file 12870_2024_5138_MOESM8_ESM.pdf]
