# Supplementary material for: Genome-wide characterisation of HD-Zip transcription factors and functional analysis of PbHB24 during stone cell formation in Chinese white pear (Pyrus bretschneideri)
Source: BMC Plant Biol. 2024 May 23;24:444. doi: 10.1186/s12870-024-05138-w (PMC11112822; doi:10.1186/s12870-024-05138-w)
Supplement: Supplementary file 9 — Supplementary Material 9 [file 12870_2024_5138_MOESM9_ESM.pdf]

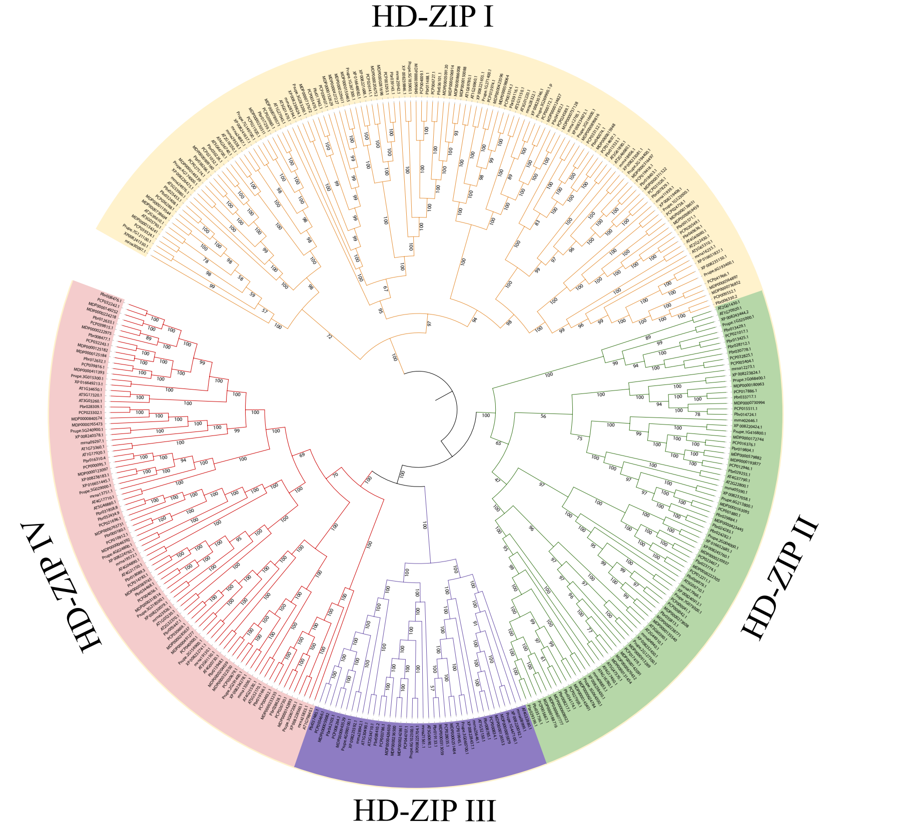

Fig. S2 Phylogenetic analysis of HD-ZIPs from 6 Rosaceae species and Arabidopsis. The tree was constructed with IQ-TREE v.1.6 software using the neighbor-joining method with 1000 bootstrap replicates. The PbHBs were clustered into 4 distinct clades, marked by curves of different colors.
